# Ulinastatin ameliorates tissue damage of severe acute pancreatitis through modulating regulatory T cells

**DOI:** 10.1186/s12950-017-0154-7

**Published:** 2017-03-20

**Authors:** Yu Pan, Haizong Fang, Fengchun Lu, Minggui Pan, Fei Chen, Ping Xiong, Yi Yao, Heguang Huang

**Affiliations:** 1 General Surgery Department, Fujian Medical University Union Hospital, No.29 Xinquan Road, Fuzhou, 350001 People’s Republic of China; 2 Department of Oncology and Hematology, Kaiser Permanente Medical Center, 710 Lawrence Expressway, Santa Clara, CA 95051 USA; 3 Fuzhou, People’s Republic of China

**Keywords:** Severe acute pancreatitis, Inflammatory responses, Ulinastatin, Regulatory T cells

## Abstract

**Background:**

Ulinastatin or urinary trypsin inhibitor (UTI) has been shown to ameliorate the inflammatory response induced by experimental severe acute pancreatitis (SAP) and hence reduce the mortality, however the mechanism of its action remains incompletely understood. We have investigated the effect of ulinastatin on regulatory T-cells (Tregs) in an established rat model of SAP.

**Methods:**

We established a rat SAP model by injecting 5% Na-taurocholate into the pancreatic duct and treated the SAP rats with ulinastatin with different dose level (5000, 10000, 30000 U/kg) through intraperitoneal injection at 0, 6 and 12 h.

**Results:**

We showed that the tissue damage of pancreas and the mortality of the SAP rats were significantly reduced by ulinastatin. We also showed that in the SAP rats the frequencies of CD4^+^ T cells and Tregs, as well as the expressions of TGF-β1, CTLA-4, and Foxp3 were decreased in the SAP animals while IL-1β, IL-10 and TNF-α were significantly increased. Treatment with ulinastatin up-regulated the proportion of Tregs in CD4^+^ T cells and the expression of IL-10, Foxp3 and CTLA-4 in the SAP rats in a dose dependence fashion, while down-regulating the levels of L-1β and TNF-α, myeloperoxidase (MPO) activity.

**Conclusions:**

Our findings suggest that ulinastatin alleviates inflammatory response and tissue damage in SAP rats by increasing the proportion of Tregs. Our study provides a new mechanism for the beneficial effect of ulinastatin in SAP rat model.

## Background

Acute pancreatitis (AP) is a common abdominal disorder characterized by systemic inflammation as well as single-organ or multi-organ failure associated with high mortality [1, 2]. The pro-inflammatory cytokines such as IL-1β, IL-10, TNFα have been shown to play an important role in the pathogenesis of AP causing tissue damage and organ dysfunction. In the course of AP, multi-organ damage caused in the early stage associated with systemic inflammatory response, followed by pancreatic necrosis in the late stage are the two most important events responsible for the mortality [3]. The intervention in the early stage by targeting inflammatory response may be an effective treatment strategy in the treatment of AP [4].

Urinary trypsin inhibitor (ulinastatin, UTI), a protease inhibitor that can be obtained from the urine of healthy people [5], was reported to have strong inhibitory activity on the pancreatic enzymes and anti-inflammatory effect on AP [6]. Maciejewski et al. [7] showed that ulinastatin administration increased the survival rate in experimental animals of AP. Furthermore, previous studies [6, 8] have shown that ulinastatin can inhibit the expression of tumor necrosis factor-α (TNF-α), and interleukin-1β (IL-1β), and increase the levels of IL-2 and IL-10. The studies on the mechanism of the anti-inflammatory effect of ulinastatin have mainly been focused on the roles of these cytokines.

Regulatory T cells (Tregs), a critical immune cell lineage, develops and matures in the thymus to regulate immune response and maintain the immune homeostasis. Conventionally, Tregs have been characterized by high expression of CD25 in CD4^+^ T cells [9]. Similarly, they express cytotoxic T-lymphocyte antigen 4 (CTLA-4) at a high level [10]. The fork-head/winged helix transcription factor p3(Foxp3) is a key nuclear transcription factor to the development and function of CD4^+^ Tregs [11, 12]. In addition to regulating immune system, there is growing evidence that Treg cells play crucial roles in controlling progressive inflammation of many diseases [13–15].

Recently, Zheng et al. [16] demonstrated that increasing the percentage of CD4^+^CD25^+^ Tregs in peripheral blood can reduce the pancreatic inflammation and mortality in a mouse model of severe AP (SAP). Another study indicated that ulinastatin can enhance immunological function and reduce the injury in SAP rats through inhibiting the apoptosis of CD4^+^ T cells [17]. Hao et al. [18] showed that UTI can attenuate inflammatory response of patients undergoing cardiopulmonary bypass by inducing the expansion of CD4^+^CD25^+^ Tregs. These studies suggested that CD4^+^CD25^+^ Tregs may play important roles in preventing the inflammatory response of AP.

In the current study, we investigated the effect and the related mechanism of ulinastatin on CD4^+^CD25^+^ Tregs in an established rat model of SAP. We studied the effects of ulinastatin on the dynamic changes of CD4^+^CD25^+^ Tregs, CTLA-4, certain nuclear transcription factor, inflammatory response, and the pathological structure in the SAP rats.

## Methods

### Animals

Male Sprague–Dawley (SD) rats, weighing 200–250 g, were obtained from the Fujian Medical University Laboratory Animal Center (Fuzhou, China) and housed in rooms with 12-h light–dark cycle for at least one week. Food and tap water were provided ad libitum. All experimental protocols were approved by the Ethical Committee for Animal Research of Fujian Medical University.

### Preparation of severe AP animal model

Severe AP was induced in male SD rats by retrograde injection of 5% Na-taurocholate (1 ml/kg body weight, Inalco S.p.A., Milano, Italy) into the pancreatic duct of the rats in accordance with the method of George Perides et al. [19]. Anesthesia was performed with intraperitoneal injection of 10% Chloral hydrate (3 ml/kg body weight; Bio Basic, Markham, ON, Canada). Afterwards, the abdominal incision was closed with sutures, and the animals received normal saline (40 mL/kg by body weight, sc). With experience, animal survival for 24 h following infusion reached almost 95%. The control groups underwent laparotomy and were subjected to insertion of a cannula into the biliopancreatic duct, but infused nothing.

### Experimental protocols

Total of 100 rats weighing 200 to 250 g were used in the study. Fifty rats were divided into 5 groups according to the animal model and treatment: control group, SAP group, SAP with ulinastatin (5000 U/kg; Techpool Biochemical Pharmaceutical Corporation, Guangzhou, China), SAP with ulinastatin (10000 U/kg), SAP with ulinastatin (30000 U/kg). In ulinastatin treatment groups, SAP-induced animals were administered with 1 ml ulinastatin through intraperitoneal injection at 0 h, 6 h and 12 h, and control group and SAP group were injected with 1 mL normal saline instead. The 0 indicates the moment when abdominal incision was closed. All rats were sacrificed at 24 h, by intraperitoneal injection of Chloral hydrate that induces lethal anesthesia. Some peripheral blood samples were collected to procure mononuclear cells and perform Flow Cytometry. The other blood samples were obtained from all previously mentioned rats for RNAs extraction and the assessment of serum amylase, lipase, and serum cytokines. Longitudinal dissected parts of the pancreas were removed and frozen in liquid nitrogen to prevent degradation for tissue MPO activity detection; other parts were fixed in 4% formaldehyde solution and then embedded in paraffin for histologic analysis. The other 50 rats, also divided into 5 groups as above, were used to evaluate the effect of ulinastatin on the mortality rate after SAP operation, respectively.

### Serum amylase analysis and ELISA

Blood samples were centrifuged at 3000 g for 10 min at 4 °C to separate the serum. The serum amylase and lipase level were measured with Olympus AV2700 automated clinical biochemistry analysis equipment (Olympus, Tokyo, Japan), according to the manufacturer’s instructions. Serum levels of interleukin 1β (IL-1β), tumor necrosis factor α (TNF-α), IL-10, and TGF-β1 were measured with a rat enzyme-linked immunosorbent assay (ELISA) kit (CUSABIO, Wuhan, China) in accordance with the manufacturer’s instructions.

### Pancreatic MPO assay and histological analysis

The pancreatic MPO assay was performed on frozen tissue using colorimetry assay kits (Jiancheng Bioengineering Institute, Nanjing, China), according to the manufacturer’s instructions. Four-micrometer sections were stained with hematoxylin and eosin (H&E) to observe the morphological changes under the light microscope. The degree of development of pancreatic lesions was evaluated according to point Spormann scale, as previously described [20]. Histological analysis was conducted in control group, SAP group, and SAP treated with ulinastatin group (30000U/kg).

### Cell isolation from the peripheral blood

The mononuclear cells were isolated from the peripheral blood by Ficoll-Hypaque gradient centrifugation (Solarbio Biotech, Beijing, China), washed twice with PBS, and kept on ice until labeling.

### Flow cytometry analysis

The expression markers on T cells from the peripheral blood were determined by flow cytometry after staining with anti-rat specific Abs conjugated with PE, FITC, or APC. The rat Abs including anti-CD4, anti-CD25, and anti-FoxP3 that were purchased from eBioscience (San Diego, CA, USA). To determine the proportion of CD4^+^ T cells in lymphocytes and CD25 expression on the surface of CD4^+^ T cells, the cells were stained with CD25-PE mAb and CD4-FITC mAb for 30 min in darkness. Concomitantly, for the detection of intranuclear Foxp3, the cells were reacted with 1 mL of freshly prepared fixation/permeabilization working solution for 2 h at 4 °C. After washing the cells with the permeabilization buffer twice, the cells were stained with anti-rat Foxp3-APC antibody for 30 min in the dark. After washing, the cells were performed on a BD Accuri C6 flow cytometer (BD Biosciences) using the FlowJo Software (Ashland, Kentucky, USA).

### Real-time polymerase chain reaction

Total RNAs were extracted from peripheral blood with TRIzol reagent (Takara, Dalian, China) followed by reverse transcription into complementary DNA (cDNA) according to the manufacturer’s instructions (Thermo Scientific, Waltham, MA, USA). Real-time quantitative polymerase chain reaction (PCR) was performed with QuantiTect SYBR Green PCR Kit (Applied Biosystems, Foster City, CA, USA) in ABI PRISM 7500 PCR instrument (Applied Biosystems) according to the manufacturer’s instructions. Glyceraldehyde-3-phosphate dehydrogenase (GAPDH) served as an internal reference. The primers were as follow: CTLA-4, 5′-TGCGGCAGACAAATGACCA-3′ and 5′-CAAAGTATGGCGGTGGGTA-3′; Foxp3, 5′-TTCTCAAGCACTGCCAAGC-3′ and 5′-GTCTCCGCACAGCAAACAA-3′; GAPDH, 5′-CTGAGTATGTCGTGGAGTCTAC-3′ and 5′-AGTCTTCTGAGTGGCAGTGATG-3′. The relative gene expression level was calculated by the 2^-ΔΔCt^ method.

### Statistical analysis

Quantitative data were expressed as the mean ± standard deviation (SD) and analyzed with a one-way ANOVA. Fisher least significant difference was used to evaluate significant differences between the groups. The survival rate was calculated as percentage of survivors at the described certain time point relative to the total number of rats that received a given treatment. The survival rate data were analyzed using the log-rank test. *P* values less than 0.05 were considered statistically significant. All statistical analyses were performed using SPSS statistical software version 19.

## Results

### Ulinastatin significantly improves survival in SAP rat model

**Fig. 1:**
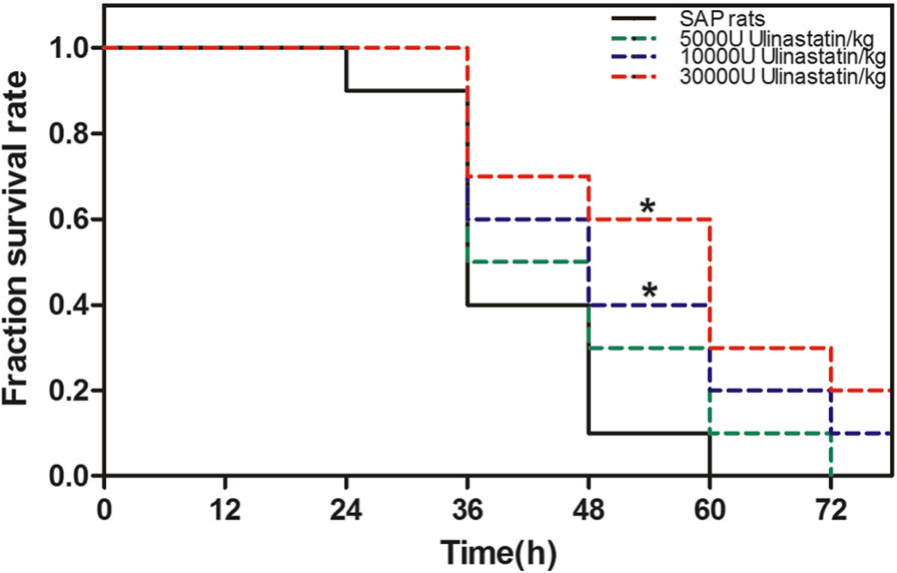
Ulinastatin treatment improves survival in SAP rats. Ulinastatin was administered immediately when SAP operation achieved and administered again in 6 h later and again in 12 h later through intraperitoneal injection. Data are shown as percent of rats surviving (*n* = 10). ^*^ *P* < 0.05 when compared with the SAP group

All rats in the sham group survived, whereas the rats that received SAP operation all died within 72 h (*P* < 0.01, Fig. 1). Intraperitoneal injection of ulinastatin (5000 U/kg) after SAP operation reduced the mortality rate (*P* > 0.05, Fig. 1). Furthermore, treatment with ulinastatin at higher dose (10000 and 30000 U/kg) significantly protected the rats from SAP (*P* < 0.05, Fig. 1).

### Effect of ulinastatin on SAP-induced enzyme production and MPO

**Fig. 2:**
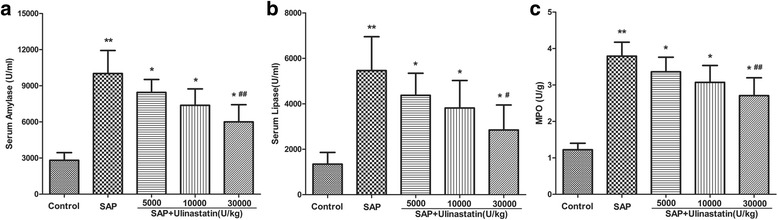
Effects of ulinastatin on SAP-induced enzyme production and MPO. **a** Serum levels of amylase. **b** Serum levels of lipase. **c** MPO activity. Data were expressed as means ± SD. *n* = 10 for each group. ^**^ *P* < 0.05 when compared with the control group; ^*^ *P* < 0.05 when compared with the SAP group; ^#^ *P* < 0.05 when compared with the SAP + Ulinastatin (5000U/kg) group; ^##^ *P* < 0.05 when compared with the SAP + Ulinastatin (10000U/kg) group

We assessed the severity of AP by measuring amylase and lipase levels, the known biomarkers of SAP. Neutrophils are known to contribute to the activation of trypsinogen to cause further damage in SAP. Therefore we next examined neutrophil infiltration by measuring MPO in pancreas homogenates. The serum amylase and lipase levels were increased significantly in the SAP group compared to the control group, indicating that SAP rat model was induced successfully (*P* < 0.01, Fig. 2a and b). Treatment with ulinastatin significantly reduced the levels of amylase and lipase in the SAP-induced rats (*P* < 0.01) compared to the untreated animals. In addition, this effect was dose dependent as ulinastatin at 30000U/kg showed pronounced effect than the 5000U/kg dose (Fig. 2a and b). Furthermore, a similar result was obtained in pancreatic MPO activities (Fig. 2c).

### Ulinastatin reduced SAP-induced tissue damage of pancreas

**Fig. 3:**
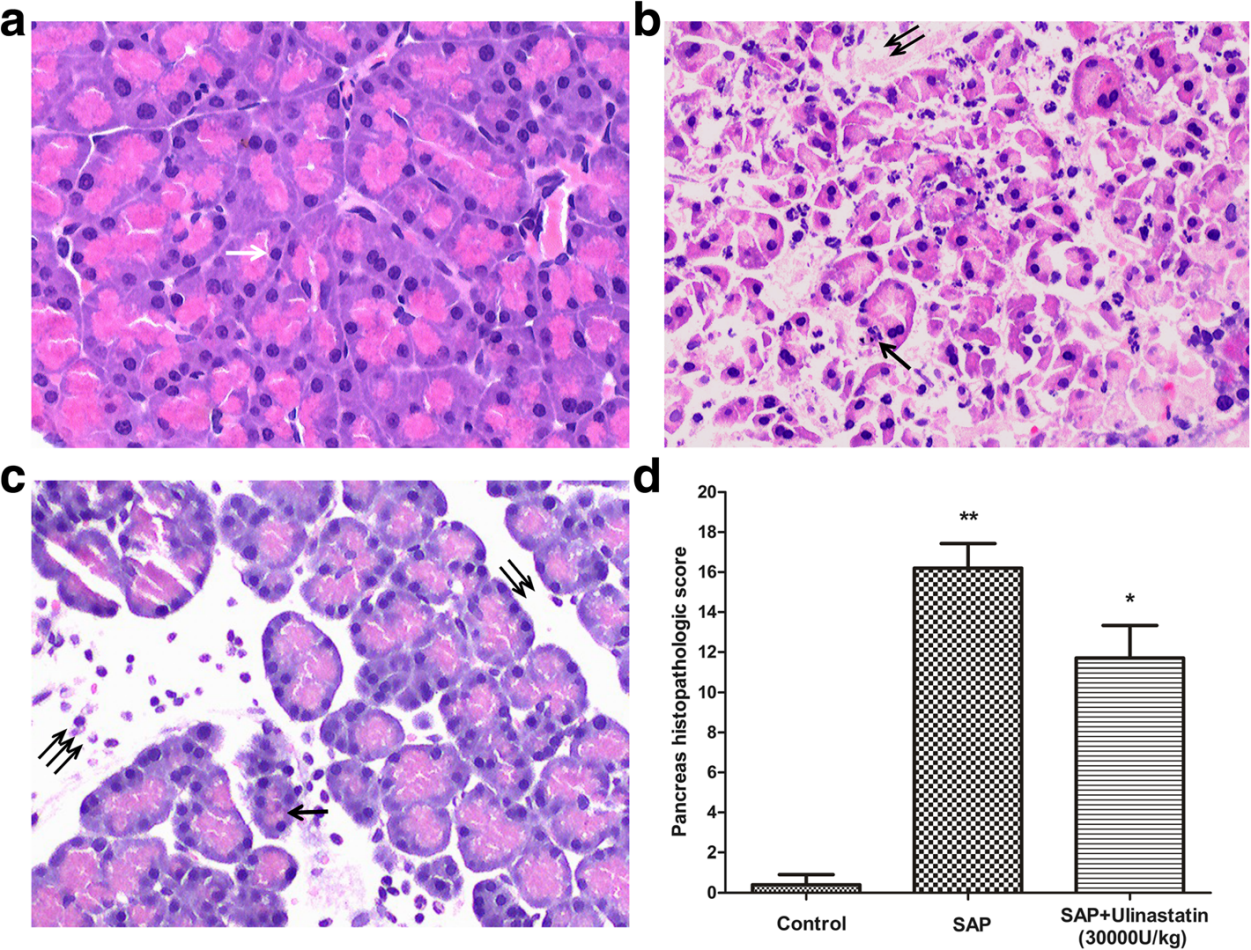
Histopathology of pancreas. **a** The pancreatic parenchyma showed typical normal architecture in control group, white arrow indicate normal acinar cell. **b** The pancreatic section showed significant destruction of structure in SAP group. **c** After ulinastatin (3000U/kg) treatment, the pancreatic damage was less severe than that in the SAP group. Single black arrows indicate glands damage and acinar cells necrosis, double black arrows indicate tissue oedema, treble black arrows indicate inflammatory cells infiltration. **d** Pancreas histopathologic score were evaluated according to point Spormann scale, and the results were expressed as means ± SE. n = 10 for each group. ^**^ *P* < 0.05 when compared with the control group; ^*^ *P* < 0.05 when compared with the SAP group. Magnification, ×200

Histologically, the pancreatic sections from the control group showed normal architecture (Fig. 3a). In contrast, the pancreatic sections from the SAP animals showed apparent destruction of normal architecture, tissue edema, necrosis of acinar cells, and infiltration of inflammatory cells, with the median point Spormann scale of 16 (*P* < 0.01, Fig. 3b and d). After treatment with ulinastatin (3000U/kg), the tissue damage was significantly reduced with the median point Spormann scale of 11 (*P* < 0.05, Fig. 3c and d).

### Effect of ulinastatin on cytokines production

**Fig. 4:**
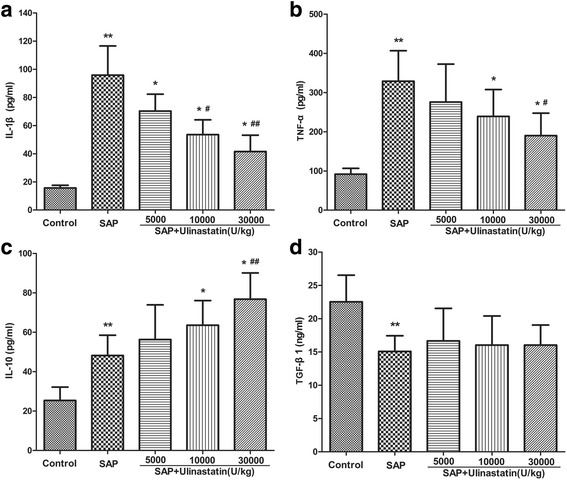
Effects of ulinastatin on serum cytokines in the SAP rats. **a** Serum levels of IL-1β. **b** Serum levels of TNF-α. **c** Serum levels of IL-10. **d** Serum levels of TGF-β1. Data were expressed as means ± SD. n = 10 for each group. ^**^ *P* < 0.05 when compared with the control group; ^*^ *P* < 0.05 when compared with the SAP group; ^#^ *P* < 0.05 when compared with the SAP + Ulinastatin (5000U/kg) group; ^##^ *P* < 0.05 when compared with the SAP + Ulinastatin (10000U/kg) group

The injury of acinar cells in acute pancreatitis is followed by a pro-inflammatory cascade leading to pancreatic necrosis and systemic inflammatory response syndrome both in human and in experimental animal models. TNF-α and IL-1β are pro-inflammatory cytokines that are known for their ability to induce systemic inflammation. In contrast to the control group, the expression of both cytokines in SAP was significantly elevated compared to the normal animals (Fig. 4a and b). Treatment with ulinastatin reduced the level of TNF-α and IL-1β in a dose-dependent way (*P* < 0.05). TGF-β1 and IL-10 are Treg-related cytokines characterized as immunosuppressive or anti-inflammatory mediators. IL-10 level was increased in the SAP animals compared to the controls while TGF-β1 showed decrease instead (*P* < 0.05). Ulinastatin treatment (10000 and 30000 U/kg) of SAP rats led to a significant increase in the serum concentration of IL-10, but no change in serum TGF-β1 level (Fig. 4c and d).

### Ulinastatin does not alter the proportion of CD4^+^ T lymphocytes in the SAP rats

**Fig. 5:**
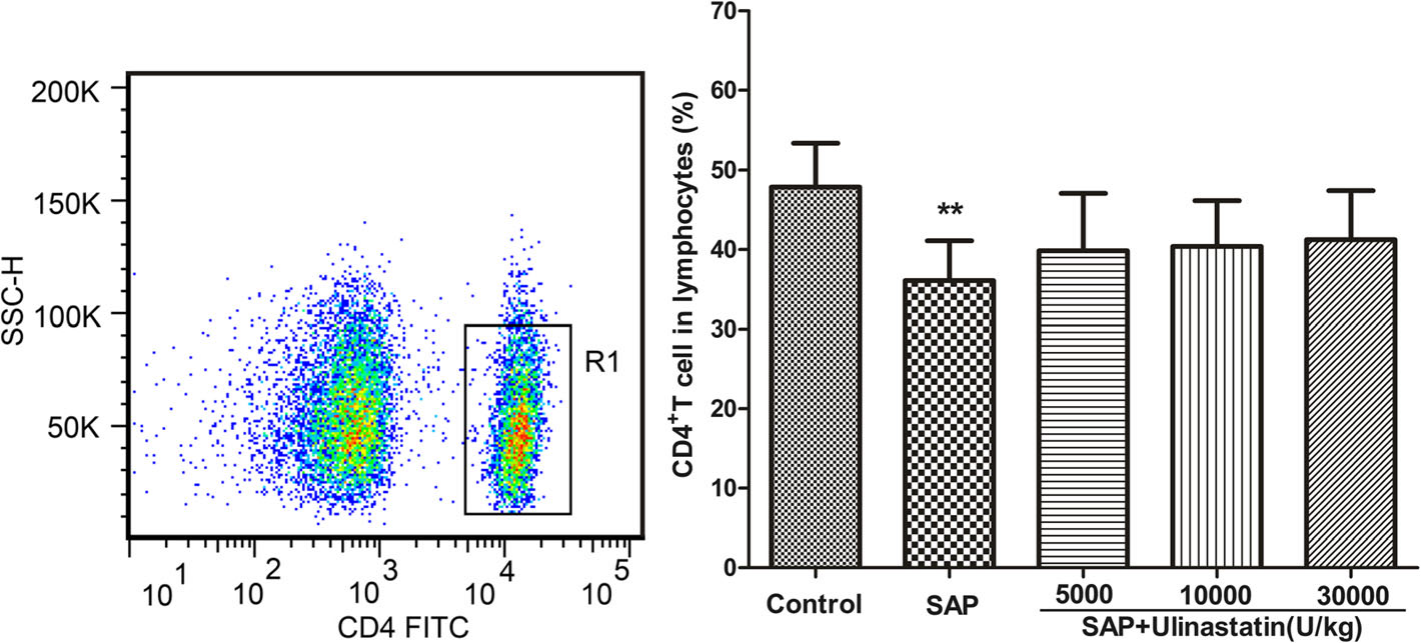
The effect of ulinastatin on circulating CD4^+^ T cells in SAP rats. R1 region represents CD4^+^ T cell in lymphocytes. The frequencies (%) of CD4^+^ T Cells were measured by using flow cytometry. Results were shown as means ± SD. n = 10 for each group. ^**^ *P* < 0.05 when compared with the control group

We explored the effect of ulinastatin on the peripheral blood CD4^+^ T cells in SAP rats by flow cytometry (Fig. 5). The SAP animals showed a lower percentage of CD4^+^ T lymphocytes compared to the controls (*P* < 0.05). There was no significant difference in the proportion of CD4^+^ T lymphocytes between SAP animals and SAP animals treated with ulinastatin.

### Ulinastatin enhances the percentage of Treg in CD4^+^ T cells in the SAP rats

**Fig. 6:**
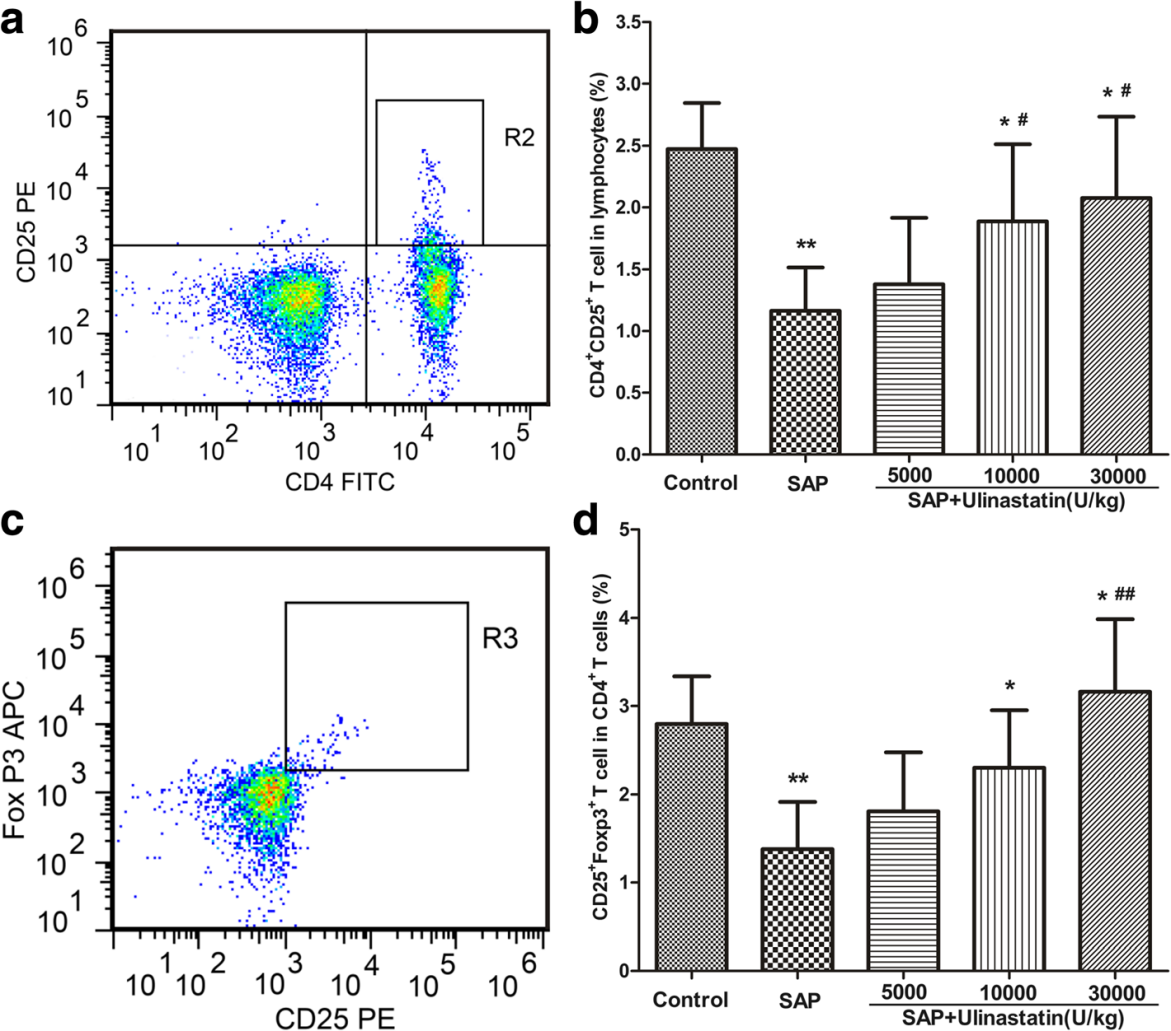
The effect of ulinastatin on Treg cells in SAP rats. **a** R2 region represents CD4^+^CD25^+^ T cells from lymphocytes. **b** The frequencies (%) of CD4^+^CD25^+^ T cells. **c** R3 region represents CD4^+^CD25^+^FoxP3^+^ T cells from CD4^+^ T cells. **d** The frequencies (%) of CD4^+^CD25^+^FoxP3^+^ T cells. Results were measured by using flow cytometry and shown as means ± SD. *n* = 10 for each group. ^**^ *P* < 0.05 when compared with the control group; ^*^ *P* < 0.05 when compared with the SAP group; ^#^ *P* < 0.05 when compared with the SAP + Ulinastatin (5000U/kg) group; ^##^ *P* < 0.05 when compared with the SAP + Ulinastatin (10000U/kg) group

We next studied the change in Tregs of the SAP animals treated with ulinastatin. The proportion of CD4^+^CD25^+^ T cells was decreased significantly in the SAP group, compared to the control group (*P* < 0.05, Fig. 6a and b). In contrast, ulinastatin (10000 and 30000 U/kg) effectively prevented the decline of CD4^+^CD25^+^ T cells (*P* < 0.05, Fig. 6a and b). Tregs were also defined as CD4^+^CD25^+^FoxP3^+^ T cells. We studied the effect of ulinastatin on the proportion of CD4^+^CD25^+^FoxP3^+^ T cells in CD4^+^ T cells, and obtained similar results (Fig. 6c and d).

### Effect of ulinastatin on Foxp3 and CTLA-4 gene expression

**Fig. 7:**
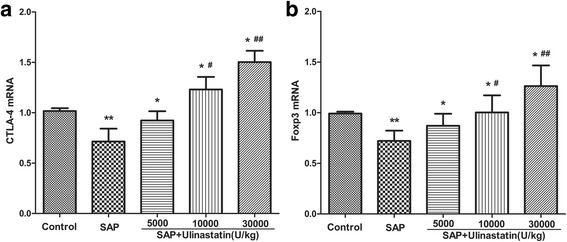
The effect of ulinastatin on mRNA expression of CTLA-4 and Foxp3 in SAP rats. **a** mRNA expression of CTLA-4. **b** mRNA expression of Foxp3. Results were expressed as means ± SD. *n* = 10 for each group. ^**^ *P* < 0.05 when compared with the control group; ^*^ *P* < 0.05 when compared with the SAP group; ^#^ *P* < 0.05 when compared with the SAP + Ulinastatin (5000U/kg) group; ^##^ *P* < 0.05 when compared with the SAP + Ulinastatin (10000U/kg) group

To explore the potential influence of ulinastatin on immunoregulatory molecules in Tregs, the expression of Foxp3 and CTLA-4 was determined with RT-PCR using lymphocytes isolated from the peripheral blood. Compared to the control group, the messenger RNA (mRNA) levels of Foxp3 and CTLA-4 in SAP group were significantly decreased (*P* < 0.05, Fig. 7). Moreover, treatment with ulinastatin further up-regulated the expression of Foxp3 and CTLA-4 in SAP rats in a dose dependence fashion, and at the 30000U/kg dose level the effect was the most pronounced (*P* < 0.05).

## Discussion

The techniques of inducing animal model of SAP include pancreatic under-capsule injection or biliopancreatic duct retrograde injection of sodium taurocholate, intraperitoneal injection of caerulein alone or injection of caerulein combined with lipopolysaccharide, ethionine diet and hypercalcemia derivation [21, 22]. The technique of Aho et al. [23] by retrogradely injecting sodium taurocholate into biliopancreatic duct through duodenal papilla puncture could more reliably induce the SAP animal model. It could reflect the patho-physiologic mechanism of acute biliary pancreatitis, a common category of clinical SAP. However, the technique causes a high damage rate of biliopancreatic duct and results in bile leakage. To overcome this problem, George Perides et al. [19] changed the way of injection by creating a small hole in the duodenal wall opposite to the papilla, followed by puncturing a cannula to complete the injection. In our study, a stable and reliable animal model was induced successfully by the modified technique of George Perides. Levels of serum amylase and lipase in SAP group were consistently elevated over the control group. Furthermore, the pathologic changes such as large necrosis area, edema and infiltration of inflammatory cells were observed in pancreatic tissue of the SAP rats.

Inflammatory cytokines including IL-1, IL-2, IL-4, IL-10 and TNF-α, are released early in the course of SAP leading to the systemic inflammatory response which represents the core problem of SAP. In the recent years, evidence has accumulated that immune reactions and immune cells like CD4^+^ T cells and Tregs play important roles in the AP pathogenesis [24, 25]. Hence, inhibiting pro-inflammatory mediators [26, 27] and regulating immune reactions [25] are important considerations in the therapy of SAP. It has been confirmed that ulinastatin prevents the inflammatory response induced by SAP [28], and evidence also indicates that ulinastatin can regulate the immunological function through these special immune cells [18, 29]. In the present study, we demonstrated that in SAP rat model, pro-inflammatory cytokines TNF-α and IL-1β and anti-inflammatory cytokine IL-10 all were increased significantly, while TGF-β was decreased. Treatment of ulinastatin led to decreased levels of TNF-α, IL-1β, and increased level of IL-10, thus attenuated the acute inflammatory response and improved the survival rate in the SAP rats. We also found that CD4^+^ T cells and Tregs were significantly decreased. The mRNA levels of CTLA-4 and Foxp3 were decreased in the SAP animals. These results indicate that not only inflammatory response, but also immune dysfunction play important roles in SAP, resulting in high mortality rate of SAP.

Tregs are pivotal to the maintenance of immune system, and key to a measured inflammatory response. Treg-related cytokines, IL-10 and TGF-β are important regulators of inflammation. During the inflammatory process, IL-10 plays the role of an anti-inflammatory cytokine, and its anti-inflammatory effect in turn regulates the function of Tregs. Some studies have demonstrated that IL-1 can affect the function of Tregs [30, 31]. Tregs express IL-1 receptor (IL-1R) and can activate p38/JNK signaling in response to IL-1 [32]. Like IL-1, TNF-α is a pleiotropic cytokine promoting inflammatory mediator cascade reaction and also impairing Treg stability and function. For instance, several studies have revealed that Tregs were functionally abnormal in patients with rheumatoid arthritis (RA), and TNF-α was the key mediator of this abnormal immune regulation [33, 34]. We found in our study that, in SAP-induced rats treated with ulinastatin, the percentage of Tregs increased significantly, in addition the expression of CTLA-4 and Foxp3 were increased, in a dose-dependence manner. Ulinastatin did not affect TGF-β1 level or the proportion of CD4^+^ T cells. Treatment with ulinastatin lessened the pancreatic tissue injury and the MPO activity, likely by enhancing the anti-inflammatory function of Tregs with the increased proportion of CD4^+^ T cells. The mechanism of ulinastatin inducing the expansion of Tregs may be through up-regulating IL-10 level and down-regulating TNF-α and IL-1β.

However, at present, it is still not clear whether the changes of these inflammatory cytokines are the primary mechanism of ulinastatin or indirect consequence of the reduced inflammatory injury after treatment with ulinastatin. In addition, the mechanism is not yet clear if ulinastatin up-regulates Tregs directly or indirectly. Also some studies showed that TNF-α can enhance the suppressive activity of Tregs [35]. The interplay between Tregs and inflammatory cytokines is complex and intimately connected and remains to be further studied.

## Conclusions

In conclusion, our study showed ulinastatin alleviates the pancreatic injury through reducing the level of MPO, pro-inflammatory cytokines, serum amylase and lipase. Our study also showed that ulinastatin up-regulated the percentage of Tregs and their anti-inflammatory function through regulating inflammatory cytokines and increasing the level of Foxp3 and CTLA-4 expression, providing evidence for a new mechanism responsible for the effect of ulinastatin in SAP.

## Abbreviations

MPO: Myeloperoxidase
SAP: Severe acute pancreatitis
IL-1β: Interleukin-1β
TNF-α: Tumor necrosis factor-α
IL-10: Interleukin-10
TGF-β1: Transforming growth factor-β1
Foxp3: Forkhead/winged helix transcription factor p3
CTLA-4: Cytotoxic T-lymphocyte antigen 4
mRNA: Messenger RNA
